# A case of sigmoid colon perforation due to segmental absence of intestinal musculature (SAIM) accompanied by vascular Ehlers–Danlos syndrome: a case report

**DOI:** 10.1186/s40792-023-01721-9

**Published:** 2023-08-02

**Authors:** Kosuke Funaki, Tomonori Akagi, Hidefumi Shiroshita, Yusuke Itai, Kiminori Watanabe, Takashi Shuto, Yoshitake Ueda, Tsuyoshi Etoh, Shinji Miyamoto, Tsutomu Daa, Masafumi Inomata

**Affiliations:** 1 Department of Gastroenterological and Pediatric Surgery, Oita University Faculty of Medicine, 1-1 Idaigaoka, Hasama-machi, Yufu, Oita 879-5593 Japan; 2 Department of Cardiovascular Surgery, Oita University, Oita, Japan; 3 Department of Comprehensive Surgery for Community Medicine, Oita University, Oita, Japan; 4 Department of Diagnostic Pathology, Oita University, Oita, Japan

## Abstract

**Background:**

Segmental absence of intestinal musculature (SAIM) is a partial defect of the intrinsic muscular layer of the intestinal tract. In this report, we describe a case of perforation of the sigmoid colon due to SAIM accompanied by vascular Ehlers–Danlos syndrome (vEDS), which was successfully treated by surgical therapy.

**Case presentation:**

A male in his 30 s was being followed up for vEDS diagnosed by genetic testing. He had undergone two major vascular surgeries, abdominal aortic artery revascularization and thoracic endovascular aortic repair for a residual dissection and enlarging abdominal aortic aneurysm. On postoperative day 11, the patient developed perforation of the sigmoid colon for which intraperitoneal lavage and drainage, Hartmann surgery, and transverse colostomy were performed. Histological findings showed no disturbance of blood flow or diverticulum but did show a defect in the intrinsic muscular layer around the perforation site, leading to the pathological diagnosis of SAIM and associated perforation of the sigmoid colon. Postoperatively, the patient had no complications and was discharged on postoperative day 18. The patient is being followed as an outpatient and has experienced no relapse.

**Conclusions:**

Both SAIM and vEDS, which may be related diseases, are associated with the presence of tissue fragility and have a high potential to cause intestinal perforation Caution should be exercised during surveillance in patients with constipation and examinations that cause increased intestinal pressure.

## Background

Segmental absence of intestinal musculature (SAIM) represents a partial loss of the intestinal intrinsic muscular layer and is a common cause of intestinal stacking and perforation in newborns and low-birth-weight infants, but it is very rare in adults. Vascular Ehlers–Danlos syndrome (vEDS) is a congenital disorder caused by a genetic mutation of type III procollagen that results in abnormal connective tissue. Thus, vEDS could also be a cause of gastrointestinal perforation. There has been no report of these diseases occurring together in an adult to date. We report the first case, to our knowledge, of an adult patient with vEDS who developed perforation of the sigmoid colon due to SAIM and underwent life-saving emergency surgery.

## Case presentation

**Fig. 1:**
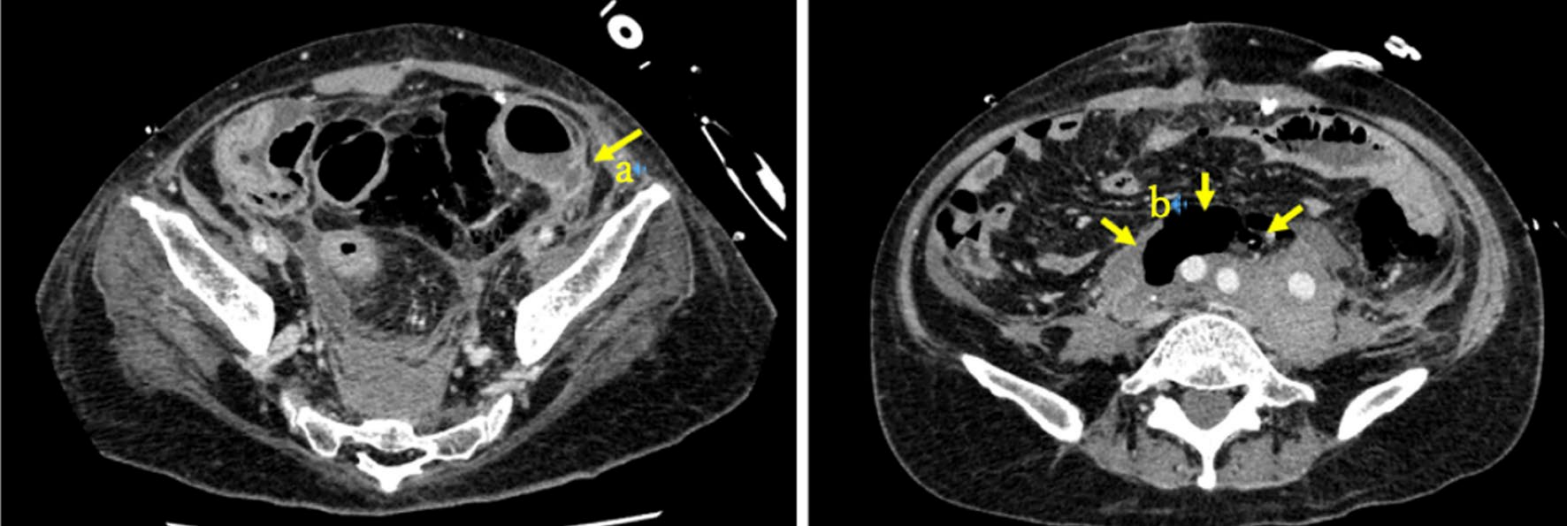
Computed tomography of the abdominal to pelvic region on postoperative day 11 shows a tear of the intestinal wall dorsal to the sigmoid colon, with a suspected perforation at the same area (arrow a). Free air is seen late in the retroperitoneum in front of the graft (arrow b)

A male in his 30 s was being followed up for vEDS diagnosed by genetic testing. He suffered residual dissection of his thoracic aorta and enlargement of an abdominal aortic aneurysm and had recently undergone two major vascular surgeries, abdominal aortic revascularization and thoracic endovascular aortic repair, in the cardiovascular surgery department of our hospital. On postoperative day 7, he complained of abdominal pain, and a computed tomography (CT) scan showed free air and ascites in the upper abdomen, which led to suspicion of perforation of the upper gastrointestinal tract, and emergency surgery was performed. A hematoma was found in the retroperitoneum, but no evidence of intestinal fluid leakage was observed. Thus, we performed only hematoma removal and drainage. On postoperative day 11 after the first surgery, abdominal pain recurred, and follow-up CT showed free air in the retroperitoneum around the vascular graft and in the left colon mesentery (Fig. 1).

Another emergency operation was performed through a midline abdominal incision, and leakage of intestinal fluid was observed near the sigmoid-descending junction of the colon, which was thought to be from a perforation in the same area. The patient had an area of mild ischemia in the descending colon to sigmoid colon, and we then suspected a disturbance of blood flow in the inferior mesenteric artery area due to the vascular graft replacement. A hematoma was observed in the retroperitoneum, and perforation and leakage of feces were observed in the sigmoid colon around the sigmoid-descending junction. The left-sided transverse colon and descending colon were resected, and a transverse colostomy was created in the left upper abdomen. The patient was discharged from the hospital after an uneventful postoperative period.

**Fig. 2:**
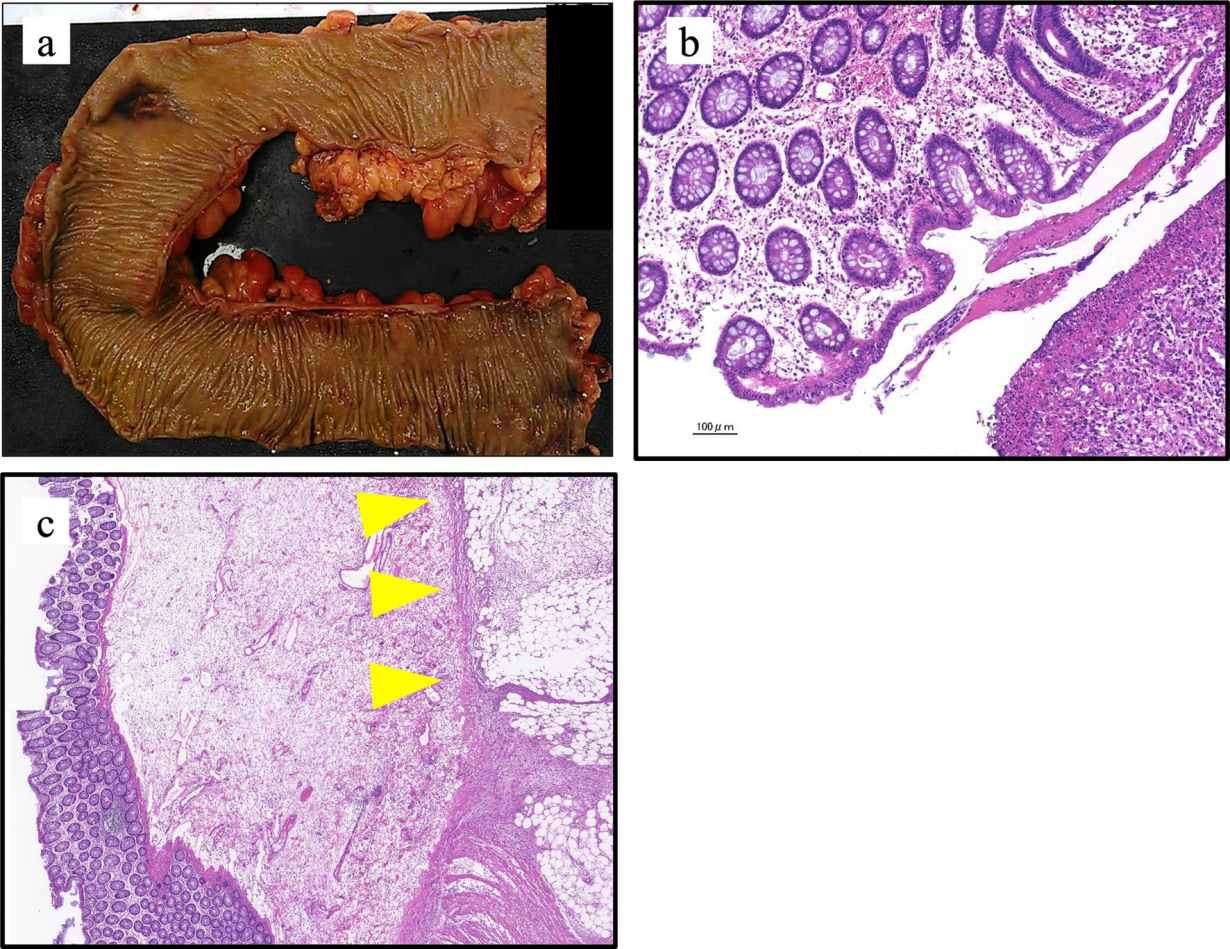
Perforation in the sigmoid colon. There was a slight loss of color from the descending colon to the sigmoid colon, suggesting impaired blood flow in the inferior mesenteric artery region after Y-graft placement (**a**), but the initial histological examination showed that the mucosa in the area of perforation was preserved, and there was little evidence of impaired blood flow (**b**). Localized thinning of the intrinsic muscular layer is also seen (arrowheads) (**c**)

Considering this patient’s history, we suspected that the perforation was caused by intestinal ischemia from vascular replacement. However, pathological examination showed that there was no evidence of either ischemia or any other causes, such as inflammatory diseases, diverticulum, or ulcer. In addition, the fact that a localized defect of the muscularis propria was observed led to the diagnosis of perforation of the sigmoid colon due to SAIM (Fig. 2).

## Discussion

There have been no reports of adult patients with coexisting vEDS who had perforation of the sigmoid colon due to SAIM and underwent surgical treatment. Herein, we reported a case in which the patient was rescued by a surgical procedure, although the preoperative diagnosis was difficult and the patient’s intestinal tissue was vulnerable.

Most frequently seen in neonates and children, SAIM is the result of a partial defect of the intrinsic muscular layer of the intestinal tract with no diverticula [1]. Darcha et al. reported the first case in an adult, in whom SIAM is very rare, in 1997 [2]. SIAM is classified into primary or secondary congenital type or acquired type [1]. Primary type results from dysplasia of the intestinal intrinsic muscular layer during fetal development and residual diverticula. It is also associated with the congenital abnormalities of biliary atresia and ventricular septal defect [3–6]. In contrast, secondary SAIM is thought to result from acute maternal hypotension in the embryonic period or hypoxia that induces ischemia and inflammation in infants with low birth weight [4, 6]. Acquired SAIM results from ischemia due to multiple surgeries or chronic constipation, among other causes [7]. Thus, ischemia due to low blood flow in the mesenteric contralateral bowel make it a frequent site of perforation [8]. However, the cause is not clearly identifiable in some patients as they have no medical history indicating a reason for the ischemia [9].

**Fig. 3:**
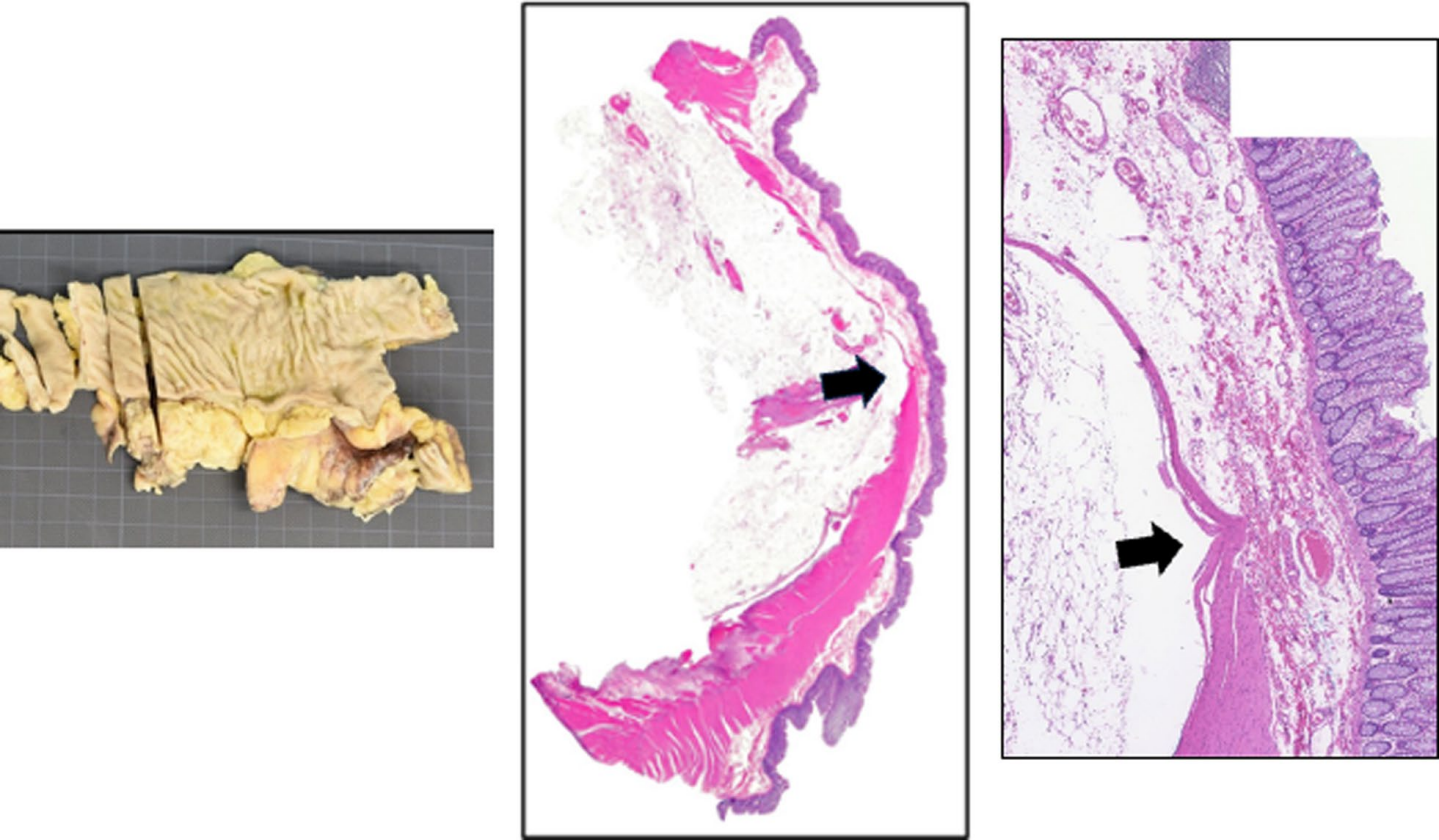
Non-perforated site in the colon. As with the perforation site, there was a defect in the intrinsic muscular layer (arrow)

There have been 20 cases of gastrointestinal perforation due to SAIM in adults, including this case. There were 9 male cases and 11 female cases and, by site, 7 cases in small intestine and 13 cases in colon (ascending colon: two cases, descending colon: two cases, sigmoid colon: nine cases). Considering past reports, gastrointestinal perforation due to SAIM is common in the sigmoid colon, but it can occur anywhere in the lower digestive tract. Thus, in the treatment strategy for SAIM, it is important to consider the possibility of residual SAIM lesions in the remaining colon. In our case, there were some localized defects of the muscularis propria besides that at the perforation site (Fig. 3), and we decided not to perform stoma closure.

vEDS is one of the six types of EDS classified according to the causative gene. Mutation of the type III procollagen gene (*COL3A1*) results in connective tissue fragility. It is a serious type of EDS that causes rupture of arteries, the intestinal tract, and internal organs, and its frequency is estimated to be 1/50,000–1/250,000. Half of the cases are inherited in an autosomal dominant manner, and half are reported to be solitary cases due to mutations and are rarely diagnosed without symptoms and a family history [10, 11].

The present patient had a sigmoid colon perforation due to SAIM and coexisting vEDS, for which there is a high risk of intestinal perforation. Only one case of intestinal perforation in a patient with vEDS and SAIM has been reported to date, that in a child [12]. Both diseases present with tissue fragility and may be related in some way. Constipation, which vEDS patients tend to have, may cause acquired SAIM, and also, the abnormality of type III collagen can cause congenital SAIM, because both diseases present with tissue fragility. This supposition will require further accumulation of cases and investigation.

There is no established treatment for the development of intestinal perforation in either SAIM or EDS [13]. In the vEDS case, there was an abnormality of type III collagen, which provides a scaffold for capillary angiogenesis and cell migration for wound healing. Thus, this can cause anastomotic leakage when a one-stage anastomosis is performed at the site of the colonic perforation [14]. Although the creation of a dividing stoma is an effective technique, surgeons should be cautious regarding colostomy closure. Among the patients with vEDS, there are several cases of gastrointestinal perforation occurring in youngsters, so these reports considered stoma closure from the viewpoints of quality of life and cosmetics. However, the risk of reperforation and suture failure was high [15]. Some authors have reported that the Hartmann procedure is safe and carries no risk of suture failure in emergency surgery in cases of generalized peritonitis due to leakage of feces [16]. Contrastingly, there are also reports that the risk of reperforation is lower with ileal colostomy or total colorectal resection with ileorectal anastomosis compared to the Hartmann procedure. Some authors even suggest that patients diagnosed as having vEDS should undergo ileal colostomy or total colorectal resection with ileorectal anastomosis as an elective surgery [17, 18]. Thus, there is currently no unified view regarding surgical treatment. Furthermore, due to the high risk of postoperative complications, some surgeons have suggested that conservative treatment be considered if the condition is such that surgery can be avoided [19]. In the present study, we chose the Hartmann operation, considering the risk of reperforation and suture failure, with the primary concern being to save the patient’s life over his general condition.

Because the risk of perforation of the residual bowel is high in both diseases, these patients must be followed with caution for conditions or procedures that increase intestinal pressure. In particular, because constipation is frequently observed in patients with vEDS, it is important to follow them with good bowel control and to consider the possibility of residual SAIM lesions in the remaining colon. Besides, it is important to care post operative paralysis of intestine, because vEDS patients often are performed operation of abdominal aorta. In our case, there was retroperitoneum hematoma, which we suspect that vEDS patients’ hemorrhagic diathesis caused, and it caused paralysis of intestine and increasing of intestinal pressure. We inserted gastric tube, but it was not enough to decrease gastrointestinal pressure.

## Conclusion

We experienced an adult case of perforation of the sigmoid colon due to SAIM accompanied by vEDS, which was treated with the Hartmann procedure. It is important to keep in mind both that SAIM is one of the less common causes of gastrointestinal perforation and the tissue fragility resulting from EDS.

## Data Availability

The data are not available for public access because of patient privacy concerns but are available from the corresponding author on reasonable request.

## Abbreviations

CT: Computed tomography
SAIM: Segmental absence of intestinal musculature
vEDS: Vascular Ehlers–Danlos syndrome
